# The development of the Be Active & Relax “Vitality in Practice” (VIP) project and design of an RCT to reduce the need for recovery in office employees

**DOI:** 10.1186/1471-2458-12-592

**Published:** 2012-08-02

**Authors:** Jennifer K Coffeng, Ingrid JM Hendriksen, Saskia F Duijts, Karin I Proper, Willem van Mechelen, Cécile RL Boot

**Affiliations:** 1 Department of Public and Occupational Health, EMGO+ Institute for Health and Care Research, VU University Medical Center (VUmc), Van der Boechorststraat 7 – C573, 1081 BT, Amsterdam, the Netherlands; 2 Body@Work TNO-VUmc, Research Center Physical Activity, Work and Health, Amsterdam, The Netherlands; 3 TNO (Expert Center Life Style), Leiden, The Netherlands

**Keywords:** Need for recovery, Relaxation, Physical activity, Environmental modifications, Group motivational interviewing, Employees, RCT

## Abstract

**Background:**

There is strong evidence to suggest that multiple work-related health problems are preceded by a higher need for recovery. Physical activity and relaxation are helpful in decreasing the need for recovery. This article aims to describe (1) the development and (2) the design of the evaluation of a daily physical activity and relaxation intervention to reduce the need for recovery in office employees.

**Methods/Design:**

The study population will consist of employees of a Dutch financial service provider. The intervention was systematically developed, based on parts of the Intervention Mapping (IM) protocol. Assessment of employees needs was done by combining results of face-to-face interviews, a questionnaire and focus group interviews. A set of theoretical methods and practical strategies were selected which resulted in an intervention program consisting of Group Motivational Interviewing (GMI) supported by a social media platform, and environmental modifications. The Be Active & Relax program will be evaluated in a modified 2 X 2 factorial design. The environmental modifications will be pre-stratified and GMI will be randomised on department level. The program will be evaluated, using 4 arms: (1) GMI and environmental modifications; (2) environmental modifications; (3) GMI; (4) no intervention (control group). Questionnaire data on the primary outcome (need for recovery) and secondary outcomes (daily physical activity, sedentary behaviour, relaxation/detachment, work- and health-related factors) will be gathered at baseline (T0), at 6 months (T1), and at 12 months (T2) follow-up. In addition, an economic and a process evaluation will be performed.

**Discussion:**

Reducing the need for recovery is hypothesized to be beneficial for employees, employers and society. It is assumed that there will be a reduction in need for recovery after 6 months and 12 months in the intervention group, compared to the control group. Results are expected in 2013.

**Trial registration:**

Netherlands Trial Register (NTR): NTR2553

## Background

In the past decades, the pressure at work has substantially increased. More than 60 percent of the employees in the EU experience that they are ‘working to tight deadlines’ [1]. Working under pressure or working with high job demands has a strong negative impact on employees’ health and well-being, leading to: cardiovascular diseases [2]; low back and neck pain [3] and; psychological distress [4]. These health problems seem to be preceded by a higher need for recovery [2,5]. The concept ‘need for recovery’ was deduced from the effort-recuperation model [6]. Need for recovery represents the short-term effects of a day at work and is described as the need to recuperate and unwind from work-induced effort. Need for recovery is especially observed during the last hours of work, or immediately after work. Typical examples of a high need for recovery are when people feel overloaded, are irritated, have decreased energy levels and disengage socially [7]. A high need for recovery can be seen as an early precursor for developing increased blood pressure [8], sleeping problems [9] and fatigue [6,10]. Need for recovery from work can be considered as an important indicator for employees who are at risk for developing health problems and motivates attention for investing in intervention programs to prevent a high need for recovery among employees.

In an effort to reduce the need for recovery, evidence has been found that physical activity is valuable in unwinding from work [11-14]. In particular, engaging in physical activity results in lower work stress [15], reduces absenteeism [16,17] and improves job satisfaction [18]. Taking time to be physically active results in an energizing effect [6], because an individual refrains from work demands and engages in activities that require other capacities than those needed in most jobs. When more time is spent on physical activity after work, and especially when it is a sports activity, the feeling of being recovered is heightened [11]. The studies previously mentioned support the claim that physical activity after work is beneficial in improving the need for recovery, disregarding the overall daily physical activity that includes active commuting and physical activity at work. Therefore, the present study will examine the effects of overall daily activity on the need for recovery in a work setting.

Relaxation is another strategy that is important for recovery. Relaxation increases one’s feeling of recovery [12,19,20]. Activities that involve relaxation are enjoyable and have a positive effect on one’s well-being [21] and self-esteem [22]. Precisely these feelings evoked by relaxation have shown to be essential for recovery [23]. Relaxation is likely to be achieved by diverting attention from work, which reverses the negative consequences of straining job demands and returns the employee to pre-stressor levels [19]. Other notable positive effects of relaxation are reduced blood pressure [24], combating an elevated resting heart rate and other symptoms of stress [25]. Having insufficient time for relaxation increases the need for recovery, which is associated with emotional exhaustion and sleeping problems [19]. Following this, it seems worthwhile to invest in an intervention program to improve daily physical activity and relaxation to reduce the need for recovery in office employees. To date, and to our knowledge, a daily physical activity and relaxation intervention in a work setting to improve the need for recovery amongst employees does not exist.

### Current project

The aim of the project is twofold: 1) to develop a physical activity and relaxation intervention to reduce the need for recovery in office employees, and 2) to evaluate the effectiveness of the developed intervention. This article describes the systematic development of the intervention based on parts of the Intervention Mapping (IM) protocol [26] (Phase 1), and the design of the effectiveness study (Phase 2).

## Methods

### Design of phase 1: Systematic development of the intervention

The intervention was developed in a systematic way, using elements of the Intervention Mapping protocol [26]. That is, a blueprint for the intervention was developed in close cooperation with employees of the Dutch financial service provider, combining scientific evidence with practice-based information of the target population. The following steps were followed: 1) needs assessment, 2) definition of program objectives, 3) selection of adequate methods and strategies to realize behavioural change, 4) program development, 5) development of a plan for pilot implementation (pilot), 6) evaluation.

#### Step 1: Needs assessment

The management of the financial service provider executed the problem analysis and designated physical activity and relaxation as a main starting point of the intervention development. The Intervention Mapping protocol was applied and information was taken from:

1. Interviews with stakeholders, with the main aim to specify current physical activity and relaxation behaviour of the target population.

2. Questionnaires, which were distributed among the target population to affirm the results of the interviews with the stakeholders, regarding physical activity and relaxation behaviour.

3. Focus-group interviews, in which the key determinants of physical activity and relaxation behaviour during work hours of the target population were identified.

Ad 1. Face-to-face interviews were held with six key persons of the Dutch financial service provider (ranging from the HR department to the IT and facility services departments). The interviews revealed that physical activity and relaxation during work hours should be more actively promoted to improve mental and physical health. Generally, it was stated that it is not approved, by colleagues and supervisors, to engage in physical activity or relaxation behaviour during work hours. During one interview, it was said that being physically active or engaging in relaxation is not something to do during work hours, but to do in your own time. In addition, the physical environment must be converted into a more physically active and relaxation friendly context.

Ad 2. The short questionnaire was completed by a convenience sample of 91 employees, who were personally approached by two of the researchers. The need for recovery was measured using the 11-items ‘need for recovery scale’ of the Dutch version of the Questionnaire on the Experience and Evaluation of Work (Dutch abbreviation VBBA) [27]. In addition, self-formulated questions about relaxation were asked. Perceptions about relaxation were measured by self-formulated questions. Employees were asked on four propositions (“I am able to engage in relaxation during work hours often”, “I am able to engage in relaxation after work hours often”, “My colleagues stimulate me to take time for relaxation”, “My supervisor stimulates me to take time for relaxation”) to which extent they agree, "totally" or "totally not agree", on a 5-point scale. In addition, a self-formulated question about facilities for relaxation was included (“At the financial service provider, there are sufficient facilities for physical activity”) with the answer category varying from "totally" to "totally not agree", on a 5-point scale. First of all, the results of the questionnaire showed that 22 percent of the employees are considered to have high scores on need for recovery and thus have a higher risk for psychological complaints [27,28]. During work hours, 39.6 % of the employees were often able to engage in relaxation, and after work hours, 81.3 % of the employees were often able to engage in relaxation. Furthermore, it was showed that 15.4 % of colleagues and 3.3 % of teamleaders support them or others to take time for relaxation. Only 8.8 % of the employees showed that there are enough facilities for relaxation. Overall, from the face-to-face interviews with stakeholders, it was concluded that the employees do not often engage in physical activity and relaxation during work hours. Furthermore, it was revealed that there are not sufficient facilities for physical activity and relaxation.

Ad 3. The results of the face-to-face interviews and the questionnaire were used as guidance for the focus group interviews. The main aim of the focus group interviews was to identify the key determinants of physical activity and relaxation behaviour during work hours. A total of five focus groups interviews were conducted with 28 employees of the Dutch financial service provider. The interviews were recorded and transcribed for analysing and coding of the data. Only those determinants that are likely to be changeable were chosen. The key determinants selected from the needs assessment for physical activity were: attitude, subjective norm, perceived behavioural control, perceived barriers and physical environment. The determinants for relaxation were: awareness, attitude, subjective norm, perceived behavioural control and physical environment. General citations from the focus group interviews regarding the determinants are depicted in Table 1. Except for physical environment, attitude, subjective norm, perceived behavioural control, determinants were related to the individual, and originate from the theory of planned behaviour [29]. Perceived barriers originate from the health belief model and focus on potential obstacles to engage in a particular health action [26]. Awareness originates from the transtheoretical model of behaviour change and entails raising awareness about a risk behaviour [26].

**Table 1:** General citations from focus group interviews regarding key determinants of physical activity and relaxation

**Determinants**	**Physical activity**	**Relaxation**
Awareness		“It is important to be aware of the advantages of relaxation”
Attitude	“Physical activity is not something you do during work hours”	“Too much relaxation inhibits fulfilment of task requirements”
Subjective norm	“Being physically active during work hours is not approved of by colleagues and supervisor”	“My colleagues state that relaxation is something that should be done after work”
Perceived behavioural control	“Increased physical activity is realized when shifting/rearranging priorities at work”	“Feeling free to choose when to relax during work hours”
Perceived barriers	“The sedentary nature of office work prohibits physical activity”	
Physical environment	“It is not an environment in which physical activity is stimulated”	“Good facilities are needed to relax”

#### Step 2: Definition of program objectives

Program objectives were formulated based on the results of the needs assessment (Step 1). The following two program objectives were chosen: (1) to increase physical activity during work hours; (2) to increase relaxation during work hours.

#### Step 3: Methods and strategies

##### Methods

In this step, for every observed determinant methods were chosen. A method is a theory-based technique to influence the determinants. Based on findings from the literature, interviews with stakeholders, the questionnaire and focus group interviews, the best methods were selected to improve physical activity and relaxation during work hours. In Table 2 and 3, the methods for the key determinants per program objective are given.

**Table 2:** Determinants, theoretical methods, strategies, tools and materials identified for improving physical activity

**Key determinants physical activity**	**Theoretical method**	**Practical strategy**	**Tools and materials**
*Attitude*	Self-regulation	MI	GMI train the trainer for teamleaders: advantages and disadvantages of changing their physical activity behaviour and the physical activity behaviour of their team members are discussed.
		MI	GMI-session with team: advantages and disadvantages of changing physical activity behaviour are discussed using a decisional balance sheet.
	Self-monitoring	MI	GMI-session with team: diary in workbook: completing logs to monitor their own daily physical activity behaviour in the last week.
*Subjective norm*	Mobilizing social support	MI	GMI-coaching for teamleaders: attention is focused on finding support among colleagues
		MI and social media platform	GMI-session with team: discussion about who can provide support. Employees can join or create physical activity groups on the social media platform. Forum on social media platform to exchange advices on physical activity.
*Perceived behavioural control*	Reinforcement/ Goal setting	MI	GMI train the trainer for teamleaders and GMI-session with team: worksheets to help extract planning goals (when, where, with whom?) for improving physical activity, and rewards for favourable outcomes are formulated. Positive feedback is given during group discussions and arguments to cope with difficult situations are discussed.
*Perceived barriers*	Self-regulation	MI	GMI train the trainer for teamleaders: exercises are aimed on intentions for changing physical activity.
		MI	GMI-session with team: exercises are aimed on overcoming barriers and enacting on intentions for changing physical activity.
	Goal setting	MI	GMI train the trainer for teamleaders and GMI-session with team: worksheets to help extract planning goals (when, where, with whom?) for improving physical activity, and rewards for favourable outcomes are formulated. Positive feedback is given during group discussions and arguments to cope with difficult situations are discussed.
*Physical environment*	Environmental changes	Facilitation of daily physical activity and relaxation	Modifications to the coffee corners, the open office environment, the meeting rooms and the entrance hall.

MI: Motivational Interviewing; GMI: Group Motivational Interviewing.

**Table 3:** Determinants, theoretical methods, strategies, tools and materials identified for improving relaxation

**Key determinants relaxation**	**Theoretical method**	**Practical strategy**	**Tools and materials**
*Awareness*	Self-regulation	MI	GMI train the trainer for teamleaders: exercises are aimed at increasing awareness for changing relaxation.
		MI	GMI-session with team: exercises are aimed at increasing awareness for changing relaxation.
	Self-monitoring	MI	GMI-session with team: diary in workbook: completing logs to monitor their own relaxation behaviour in the last week.
*Attitude*	Self-regulation	MI	GMI train the trainer for teamleaders: advantages and disadvantages of changing their relaxation behaviour and the relaxation behaviour of their team members are discussed.
		MI	GMI-session with team: advantages and disadvantages of changing relaxation behaviour are discussed using a decisional balance sheet.
	Self-monitoring	MI	GMI-sessions with team: diary in workbook: completing logs to monitor their own relaxation behaviour in the last week.
*Subjective norm*	Mobilizing social support	MI	GMI-coaching for teamleaders: attention is focused on finding support among colleagues
		MI and social media platform	GMI-session with team: discussion about who can provide support. Employees can join or create relaxation groups on the social media platform. Forum on social media platform to exchange advices on relaxation.
*Perceived behavioural control*	Reinforcement/ Goal setting	MI	GMI train the trainer for teamleaders and GMI-session with team: worksheets to help extract planning goals (when, where, with whom?), and rewards for favourable outcomes are formulated. Positive feedback is given during group discussions and arguments to cope with difficult situations are discussed.
*Physical environment*	Environmental changes	Facilitation of daily physical activity and relaxation	Modifications to the coffee corners, the open office environment, the meeting rooms and the entrance hall.

MI: Motivational Interviewing; GMI: Group Motivational Interviewing.

Methods as self-regulation, self-monitoring, mobilizing social support, reinforcement, goal setting and environmental changes will be applied for improving the determinants of daily physical activity and relaxation. The methods self-regulation and self-monitoring will be applied to influence the determinants attitude, perceived barriers and awareness. Self-regulation refers to: “an active, iterative process, of goal setting, choosing strategies, self-observation, making judgements based on observation, reacting appropriately in the light of one’s goals and revising one’s strategy accordingly” (26). The determinant attitude will also be influenced by self-monitoring. Self-monitoring is a process in which an individual keeps track of particular behavioural patterns [30]. For influencing the subjective norm, mobilizing social support is a helpful aid in the change process [31,32]. It is important that an individual searches for support in his of her immediate environment, e.g., partner, close relatives and colleagues. To enhance perceived behavioral control, reinforcement is of importance. Reinforcement, e.g., by formulating rewards, plays a critical role in obtaining favorable outcomes. Another aid in influencing perceived behavioural control is by means of goal setting. Goal setting involves the formulation and prioritization of goals [31,32]. To improve the physical environment, changes in the environment must be made that stimulate physical activity and relaxation.

##### Strategies

The next step was to select strategies for the methods and key determinants. A strategy operationalizes the intervention methods and the strategies are given in Table 2 and 3. Based on previous steps, gathered literature, the advice of the project group and feedback from experts in the field, the following strategies were considered to be applicable to target determinants and methods: (A) Group Motivational Interviewing (GMI) consisting of a GMI-training (train the trainer for teamleaders), GMI-coaching and GMI-sessions. The GMI-sessions will be supported by a social media platform; and (B) environmental modifications. Motivational interviewing (MI) is a counseling style that stimulates behavioural change by focusing on exploring and resolving ambivalence [33]. First of all, it was decided to adjust individual MI to group MI. There is increasing support that individual MI can be effectively adjusted to a group format [34-39], defined as Group Motivational Interviewing (GMI), which consists of concepts of self determination theory [40] and individual MI [33]. A group setting has several benefits, e.g., sharing experiences, providing feedback and giving support [39]. GMI helps to create an autonomous supportive environment, in which engaging in daily physical activity and relaxation can be encouraged [35]. Following this, with applying GMI, the method of mobilizing support will be carried out (Table 1). During the GMI-coaching and GMI-sessions, attention will be paid to who can provide support. The web-based social media platform plays a role in mobilizing social support by means of the forum and creating and/or joining activities groups. The other methods that target the key determinants are carried out using the GMI-training (train the trainer for teamleaders), GMI-coaching and GMI-sessions, such as improving self-regulation, encouraging self-monitoring, giving reinforcement and stimulating goal setting (Table 2 and 3). To illustrate, self-regulation is improved by means of the decisional balance sheet, which discusses the advantages and disadvantages of changing behaviour. To enhance self-monitoring, employees will be asked to complete logs for a week about their daily physical activity and relaxation behavior. Furthermore, during the GMI-training and GMI-sessions, a worksheet will be filled in by stating goals with the subsequent rewards. In a group discussion, the goals and rewards will be discussed. Another strategy is to make changes in the environment. It has been shown that environmental modifications such as providing exercise space and equipment, red line routes to promote walking during lunch hours, posters to promote stair use and a walking track are effective [41]. Furthermore, research has demonstrated that, for example, changes in office layout and/or office furniture can positively influence employee's attitudes [42], job satisfaction and performance [43-46]. Another study has shown that changing the interior of the meeting room improved comfort for the employee and has helped the employee to focus on the meeting [47]. Another example of an effective strategy was placing standing tables in the office environment, showing that stand-up meetings are held in a more timely manner than sit-down meetings [48]. Painting office walls can be beneficial as well; it has shown to positively influence productivity levels [49]. Since concentration is hindered by telephone noise and people chatting in the background [50], any modifications to decrease office noise can be helpful [51]. Following this line of reasoning, it can be hypothesized that changing the work environment has an impact on the two key variables under study, i.e., physical activity and relaxation, by providing an environment that promotes daily physical activity and relaxation.

#### Step 4: Program development

##### A. Group Motivational Interviewing (GMI)

GMI will be delivered by the teamleaders of the departments, allocated to the GMI-intervention. They will be invited for a two-days training in GMI, which will be conducted by a GMI-professional. After completing the training, the educated teamleaders will conduct three GMI-sessions of 90 minutes each, within a period of six weeks (i.e., 0, 3 and 6 weeks), with the employees in his/her team. A booster session, also given by the teamleader, will be scheduled two months after the final session. The time schedule was decided upon in consultation with the stakeholders and project group. In the literature, no advice was found regarding an optimal schedule for the GMI-sessions. All GMI-sessions take place during work hours. The main aim of these sessions is to stimulate physical activity and relaxation. During the GMI-sessions, the teamleader will focus on fostering a readiness to change, and on lowering resistance and ambivalence towards changing daily physical activity and relaxation behaviour. For each session, materials will be developed, e.g., a manual with theoretical information and detailed instructions, which can be used by the teamleader to supervise the sessions. Furthermore, a workbook for the employees will be developed in which all the exercises for the GMI-sessions are given. During the program, the teamleaders will have two GMI-coaching meetings of 90 minutes each, supervised by a GMI-professional, to share experiences with each other.

### Social media platform

In this study, the GMI-sessions will be supported by a web-based social media platform. The platform is meant for employees allocated to the GMI-intervention arms of the study. The main goal of this platform is that employees can create or join physical activity and relaxation groups, such as playing on a dartboard, walking during lunch hours, engaging in physical exercise and taking part in a book club. Furthermore, the employees will be able to find information on this platform, which will be frequently updated by the primary researcher of the VIP program.

#### B. Environmental modifications

The aim is to facilitate daily physical activity and relaxation in the work environment, by changing the coffee corners, the open office environment, the meeting rooms and the entrance hall. At six departments, several “Be Active & Relax” zones will be created: (1) the VIP Coffee Corner Zone will be modified by adding a bar with bar chairs and a giant wall poster (a poster visualizing a relaxing environment, e.g., wood, water and mountains); (2) the VIP Open Office Zone will be modified by introducing exercise balls and curtains to divide desks in order to reduce background noise; (3) the VIP Meeting Zone will be modified by placing a standing table and a giant wall poster (a poster visualizing a relaxing environment e.g., wood, water and mountains); and (4) in the VIP Hall Zone, a table tennis table will be placed and lounge chairs will be introduced for informal meetings. In addition, footsteps will be placed on the floor in the entrance hall to promote stair walking. All these environmental modifications will be promoted by a banner in the VIP Coffee Corner Zone. This banner will visualize all the environmental modifications and their location at the department. Furthermore, the teamleaders and employees will receive information by email about the environmental modifications.

#### Step 5: Development of a plan for pilot implementation

The two-days GMI-training, three GMI-sessions with employees, and materials were all pilot tested with intended users. The pilot GMI-training was executed with seven teamleaders of a related division of the financial institute. The pilot of the training was evaluated at the end of day two by means of a group discussion about strengths and limitations. This resulted in several adaptations in the format of the GMI-training and the manual. In addition, one teamleader executed three GMI-sessions with six employees. Again a group discussion about strengths and limitations was held and this resulted in several revisions of the workbook and format of the three GMI-sessions. Due to time and budget constraints, the environmental modifications were not pilot tested.

#### Step 6: Evaluation

A description of the evaluation of the study on the effectiveness of the intervention is presented in the following sections (phase 2).

### Phase 2: A description of the evaluation on the effectiveness of the intervention

#### Study design

The effectiveness of the intervention program will be investigated in a trial using a modified 2x2 factorial design. The two factors are the GMI intervention and the environmental modifications, of which the environmental modifications will be stratified and GMI will be randomised on department level (Table 4). This will result in four research arms, i.e.: (1) GMI and environmental modifications; (2) environmental modifications only; (3) GMI only; (4) no intervention (control group).

**Table 4:** Modified 2 X 2 factorial design

		**Environmental modifications #**	
		**YES**	**NO**
GMI*	YES	1	3
	NO	2	4

GMI: Group Motivational Interviewing. *GMI will be randomized; # Environmental modifications will be stratified.

**Figure 1:**
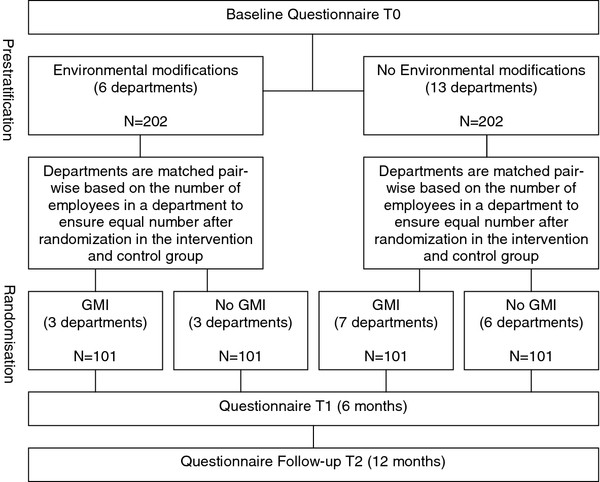
Design VIP program.

Environmental modifications will be applied to six departments of the Dutch financial service provider. Two strata will be created: (1) departments with environmental modifications (N = 6) and (2) departments without these modifications (N = 13). Within these strata, the departments will be randomized to GMI or no GMI by means of tossing a coin. Each department consists of several teams, and each team includes a teamleader and his/her employees. Due to practical reasons in only 6 departments environmental changes will be made. Therefore, departments will be matched pair-wise, based on the number of employees in a department, to ensure equal numbers after randomization in the intervention and control group. The design of the VIP program is visualized in Figure 1.

Employees who have given informed consent will complete questionnaires at baseline (T0), at 6 months (T1), and at 12 months (T2) follow-up. The study protocol has been approved by the Medical Ethics Committee of the VU University Medical Center (Amsterdam, The Netherlands).

#### Study population and recruitment

The sample will consist of office employees (≥18 years), working at the Dutch financial service provider. For this study, teamleaders and their employees from 19 departments are eligible to participate. An exclusion criterion will be ‘being on sick leave for more than four weeks’. To successfully accomplish the VIP program, a top-down communication approach will be used, starting with the management, continuing towards the teamleaders. At an explanatory meeting, teamleaders will receive an envelope with information on the study and the baseline questionnaire, including an informed consent form. Next, all employees from the departments will be invited to participate in the study, i.e., they will receive an invitational letter, information on the study, and the baseline questionnaire, including an informed consent form. By signing the informed consent, teamleaders and employees will give their consent for participation in the study and for obtaining additional data. That is, data on sick leave, salary and the duration of employment will be obtained through the HRM department.

#### Outcome measures

Primary outcome measure of the study is need for recovery, which will be assessed using the Need for Recovery after Work scale [27] by questionnaires at baseline, 3, 6, 9 and 12 months follow-up. This Dutch version of the Questionnaire on the Experience and Evaluation of Work (Dutch abbreviation: VBBA) consists of eleven dichotomous items (yes/no). The need for recovery has been evaluated among 601 employees from various organisations in the Netherlands, and has shown satisfactory validity (r = 0.65) and reliability (r = 0.87) [27].

Secondary outcome measurements of this study are daily physical activity, detachment and relaxation.

 – Daily physical activity, which will be assessed at baseline, 6 and 12 months follow-up by the Short Questionnaire to Assess Health Enhancing Physical Activity (SQUASH) [52]. This questionnaire assesses the duration, frequency, intensity of active commuting, physical activity at work, sedentary time at work and at home, activity in leisure time, household activities, and sport. The validity (r = 0.45) and reproducibility (r = 0.58) of the SQUASH are comparable to other physical activity questionnaires [53].

 – Physical activity and sedentary behaviour, which will be measured objectively in a random sample of about 80 participants, equally divided over the four study arms (n = 20 per arm). This subsample will be asked to wear an accelerometer (Actigraph) during a period of seven days at baseline, 6 and 12 months follow-up. On all days of the week, these participants will be asked to fill out a short questionnaire (diary), reporting the exact wearing times (i.e., the time at which the Actigraph was put on and off) and the times of leaving and arriving at home and at work (i.e. to determine the exact wearing time at work and during leisure time).

 – Detachment and relaxation, which will be assessed at baseline, 6 and 12 months follow-up by the detachment and relaxation questionnaire. This questionnaire is derived from the recovery experience questionnaire, which is developed by Sonnentag et al..[19], and which has shown satisfactory reliability (r = 0.84) and reasonable validity (r = 0.46). A Dutch version of this questionnaire has shown high reliability (r = 0.92) in a pilot study by Bloom et al [54]. This scale was adapted to a work context, starting each item with “During a break at work…”, instead of “During time after work…” as written in the original questionnaire of Sonnentag et al.[19]. The detachment and relaxation questionnaire consists of eight items and lists items such as “I forget about work”, “I don’t think about work at all”, “I kick back and relax” and “I do relaxing things”.

#### Other outcome measurements

 – Exhaustion will be assessed at baseline, 6 and 12 months follow-up by means of a subscale of the Oldenburg Burnout Inventory (OLBI) [55]. The OLBI consists of eight items about exhaustion and disengagement. It has shown reasonable validity (r = 0.52) and satisfactory reliability (r = 0.80) in different occupational groups [55].

 – Absenteeism data will be collected by questionnaires at 3, 6, 9 and 12 month’s follow-up through self-report, as well as from company records. The World Health Organization Health and Work Performance Questionnaire (WHO-HPQ), a self-report measure concerning absenteeism and presenteeism, will be used [56, 57]. To illustrate, participants will be asked to give ‘the number of days missed due to mental or physical health problems, during the last 4 weeks’. Furthermore, the PROductivity and DISease Questionnaire (PRODISQ) will be used [58, 59].

 – Work performance will be assessed at baseline, 6 and 12 months follow-up with the Individual Work Performance Questionnaire (IWPQ)[60]. The IWPQ consists of 43 questions in four subscales: task performance, contextual performance, adaptive performance, and counterproductive work behaviour. The IWPQ is a newly developed instrument based on a review of the work performance literature, existing work performance questionnaires, and expert opinions [60].

 – Work engagement will be measured at baseline, 6 and 12 months follow-up, using the validated Utrecht Work Engagement Scale (UWES), which assesses vitality (6 items), dedication (5 items) and absorption (6 items). The psychometric properties of this questionnaire have been tested and results indicated an acceptable internal consistency of vitality (r = 0.68-0.80), dedication (r = 0.91) and absorption (r = 0.73-0.75)[61, 62].

#### Prognostic factors

Sociodemographics, such as age, sex, working hours per week, education level, and body height will be assessed at baseline. Body weight will be assessed by self-report at baseline, 6 and 12 months follow-up.

Job characteristics will be assessed only at baseline, with 30 items of the validated Dutch version of the Job Content Questionnaire (JCQ) [63], which includes the subscales job demands, skill discretion, decision authority, decision latitude, supervisor and colleague support and job insecurity. The JCQ has shown satisfactory reliability (r = 0.72) and is widely used [63]. The JCQ is derived from the Job Demands-Control-Support model of Karasek and Theorell [64].

Determinants of behavioural change for physical activity and relaxation will be examined by means of the determinants lifestyle behaviour questionnaire (DLBQ), which has been validated by Lakerveld et al. [65]. The key determinants found during the need assessment are attitude, subjective norm, perceived behavioural control and physical environment and will be measured at baseline. A study of Lakerveld et al. demonstrated that the DLBQ is able to measure determinants that precede the intention to change physical activity in adults [65] and these items were revised in consultation with the developer of the DLBQ and the project group to measure relaxation as well.

General health and mental health will be measured by items of the Dutch validated version of the Rand-36 [66], which has shown satisfactory reliability (r = 0.83) and reasonable validity (r = 0.49). General health perceptions will be measured by asking employees to give an indication on a 6-point scale how they perceive their health and to indicate on four propositions (e.g. “I fall more easily ill than others”) to which extent they “totally agree” or “totally disagree”. To assess mental health, employees will be asked to indicate on a 6-point scale how often they felt full of life, worn out, tired and full of energy, during the past four weeks [66]. At 6 and 12 months follow-up, only the four items of vitality from the Rand-36 will be measured.

To measure stress, the short form of the Perceived Stress Scale (PSS; 4 items) will be used [67]. PSS-4 is considered to be sound, but it has a rather low internal reliability (r = 0.60). Both the 10- and 14-item self-report of PSS have higher satisfactory reliability and validity [67]. However, the PSS-4 was chosen for this study, because it is not meant as a diagnostic instrument, but to measure general stress levels at baseline.

Sleep quality will be assessed by the Jenkins Sleep Problems Scale (JSPS) [68]. This scale contains four items, i.e. trouble falling asleep, continue sleeping, waking up several times at night and waking up feeling tired and worn out. The scale has shown an acceptable test-retest reliability (r = 0.59) and an internal consistency of r = 0.63 [68]. Both PSS 4 and the JSPS will be measured at baseline only.

#### Economic measurements

Intervention costs that will be measured include the costs of GMI (i.e., employment costs of GMI-professionals/teamleaders, facilities, and materials), the social media platform (i.e., website development, hosting, and maintenance), modifications to the office building, and printed materials. Since GMI takes place during work hours, the costs of lost productivity due to the intervention will be included as well. Teamleaders will record the frequency and duration of group meetings as well as the attendance rates of their employees. Intervention costs will be measured and valued using a micro-costing bottom-up approach [69].

Health care costs that will be measured will include care by the general practitioner, allied health care, medical specialist, complementary and alternative medicine, hospitalisation, and (prescribed) medications. Data on resource use will be collected on a three-monthly basis, using retrospective questionnaires. Dutch standard costs will be used to value health care utilization [70]. If these are not available, prices set by professional organizations will be used. Medication use will be valued using unit prices provided by the Dutch Society of Pharmacy [71].

Productivity-related costs that will be measured include workplace productivity loss, as described above. Workplace productivity losses (i.e., absenteeism and presenteeism) will be valued using the gross annual salaries including holiday allowances and premiums of the employees as well as standard mean labor costs of the Dutch population.

Participant costs that will be measured include self-reported costs related to sports activities (e.g., membership fees and sports equipment costs), since the intervention stimulates employees to engage in daily physical activity and relaxation. These costs will be collected on a three monthly basis.

### Statistical analysis

#### Effect evaluation

Multilevel regression analyses will be performed regarding the effectiveness of the intervention on need for recovery, and on secondary outcome measures at 6 months (short-term) and 12 months (long-term) follow-up and adjusting for the baseline values of the outcome measure. In addition, a Generalised Estimated Equations (GEE) analysis will be performed for the long-term effects (baseline to 12 months). All analyses will be performed according to the intention-to-treat principle. In addition, per-protocol analyses will be performed on those employees who have completed 50 % of the GMI-sessions, logged into the social media platform and made use of the environmental modifications at least once. For all analyses, a two-tailed significance level of p < 0.05 will be applied. The multilevel analyses will be performed with MlwiN 2.0; linear and logistic regression analyses will be performed with SPSS 19.0 (SPSS Inc. Chicago, Illinois, USA).

#### Process evaluation

A process evaluation will be performed, using the framework of Steckler and Linnan 2002 as a basis [72,73] to gain insight into the factors (context, recruitment, fidelity, reach, dose delivered, satisfaction and dose received) that influence the effectiveness of the VIP program.

#### Economic evaluation

The economic evaluation aims to determine the cost-effectiveness of the intervention compared with usual care, from both the societal and employer’s perspective. Also, cost-benefit will be determined from the employer’s perspective. The follow-up will be one year, similar to the trial, so discounting will not be needed [74]. Analyses will be performed according to the intention-to-treat principle. In the main analysis, missing data will be imputed using multiple imputation techniques [75]. Sensitivity analyses will be done to assess the robustness of the results. Total societal and employer’s costs will be estimated, and compared between the intervention and control group. The 95 % confidence intervals will be estimated using approximate bootstrap confidence (ABC) intervals [76]. Societal costs will include all cost measures described in the study parameters section. From the employer’s perspective, only costs relevant to the employer will be included (i.e. intervention, and productivity-related costs). For the cost-effectiveness analysis (CEA), incremental cost-effectiveness ratios will be calculated by dividing the difference in costs between both groups to their difference in effects on the primary outcome measures (societal perspective), and outcome measures relevant to the company (employer’s perspective). Bootstrapped cost-effect pairs will be graphically presented on cost-effectiveness planes [77]. Cost-effectiveness acceptability curves will be generated, showing the probability for cost-effectiveness of the intervention at different ceiling ratios. Also, a cost-benefit analysis (CBA) will be performed in which the incremental intervention is compared to the incremental productivity-related costs.

#### Sample size calculation

A sample size calculation has been performed, based on the main outcome measure, i.e. need for recovery, measured by the Need for Recovery after Work scale of the Dutch VBBA questionnaire [27]. The mean need for recovery score is 27.30 (SD = 29.75), according to the validation study of Van Veldhoven et al. [27]. Minimal relevant difference on the need for recovery scale, with a range from 0 to 100, is set at 12, based on studies by De Croon et al. [78] and Kuijer et al. [79]. An intraclass correlation (ICC) of 0.025 is assumed, based on previous studies showing that worksite level ICC’s for health-related outcomes are generally small [80-82]. By using the ICC for departments, the power analysis revealed that a sample size of 305 employees will be needed, with a power of 80 % and an alpha of 5 %. Finally, accounting for a loss to follow-up of 25 % in twelve months, the entire study population will need to consist of 404 employees.

## Discussion

This article aims to describe (1) the development and (2) the design of the evaluation of a daily physical activity and relaxation intervention to reduce the need for recovery in office employees. The systematic development of the intervention was based on parts of the IM protocol, involving key persons and the target population of the financial service provider. As a result of the needs assessment, the following two program objectives were drawn up: (1) to increase daily physical activity at work, (2) to increase relaxation at work. Key determinants for daily physical activity and relaxation were identified for the target population. Methods and strategies were chosen to target these determinants. The VIP program consists of GMI supported by a social media platform, and environmental modifications.

### Strengths and limitations

An important indicator for employees who are at risk for developing health problems is a high need for recovery from work [2,5]. Consequently, one of the main strengths of the VIP program is that it pioneers in combining a physical activity and relaxation intervention to improve the need for recovery in office workers. Another strength is that the VIP program has been developed and will be implemented in a real-life setting. It was custom-made together with management and employees of the participating company with IM as a supportive tool. Herewith, this pragmatic trial will support generalizability [83]. That is, we believe that customization will improve compliance, in current and future work settings, and that this will contribute to the effectiveness of the program. Furthermore, the effectiveness will be evaluated in a RCT, using a modified 2x2 factorial design with a long term follow-up. In addition, the VIP program contains elements that will target both the individual behaviour and the environment. Ecological models state that healthier behaviour can be achieved by influencing various levels, such as the intrapersonal level, the interpersonal level, the organizational level and the community. Combining these levels is necessary to achieve behavioural change [84]. Moreover, a combined intervention is likely to demonstrate effects over extended periods of time [85].

Apart from the above strengths, there are some limitations. As the program has been developed with the target population, it might be that the current VIP program is only applicable for this specific target population and external validity may be limited. Second, contamination may occur when control participants enter the departments with environmental modifications on a frequent basis. When evaluating the effects, this must be considered. Chances of contamination with GMI are minimal, because the GMI-sessions will be only given for those in the GMI-intervention group. Finally, logistical and organizational factors contributed to our decision to stratify environmental modifications and to randomise GMI.

## Conclusion

The development of the intervention by using parts of the IM protocol has resulted in a physical activity and relaxation program to reduce the need for recovery in office employees. More knowledge on the need for recovery may help to improve programs on work ability, return to work and employability of employees. Results on cost-effectiveness will show whether the benefits of improving need for recovery in a work setting outweigh the expenditures. If the VIP program proves to be (cost-) effective, the financial service provider and society will benefit from a program that will reduce the need for recovery.

## Competing interests

The authors declare that they have no competing interests.

## Authors’ contributions

JC wrote the initial protocol. IH, SD, KP, WvM and CB were involved in the development of the VIP program. All authors have read and approved the final version of the manuscript.

## Pre-publication history

The pre-publication history for this paper can be accessed here:

http://www.biomedcentral.com/1471-2458/12/592/prepub
